# Maximum Swing Flexion or Gait Symmetry: A Comparative Evaluation of Control Targets on Metabolic Energy Expenditure of Amputee Using Intelligent Prosthetic Knee

**DOI:** 10.1155/2018/2898546

**Published:** 2018-11-21

**Authors:** Wujing Cao, Weiliang Zhao, Hongliu Yu, Wenming Chen, Qiaoling Meng

**Affiliations:** ^1^Rehabilitation Engineering and Technology Institute, University of Shanghai for Science and Technology, Shanghai 200093, China; ^2^Shanghai Engineering Research Center of Assistive Devices, Shanghai 200093, China

## Abstract

**Background:**

The metabolic energy expenditure (MEE) was the most important assessment standard of intelligent prosthetic knee (IPK). Maximum swing flexion (MSF) angle and gait symmetry (GS) were two control targets representing different developing directions for IPK. However, the few comparisons based on MEE assessment between the MSF and GS limited the development of the IPK design.

**Objectives:**

The aim of the present work was to find out the MEE difference of amputees using IPK with control targets of MSF and GS and determine which target was more suitable for the control of IPK based on the MEE assessment.

**Methods:**

The crossover trial was designed. Six unilateral transfemoral amputees participated in the study. The amputees were assessed when wearing the IPK with different control targets, namely, the maximum swing flexion angle and gait symmetry. The oxygen consumption analysis during walking at different speeds on a treadmill was carried out.

**Results:**

All subjects showed increased oxygen consumption as walking speed increased. However, no statistically significant differences were found in oxygen consumption for different control targets. The ANOVA test showed that the overall effects of the control targets of the prosthetic knee on oxygen consumption were not significant across all walking speeds.

**Conclusions:**

The control targets of MSF and GS showed no significant differences on MEE in above-knee amputees using IPK. From perspective of amputee's metabolic costs, either maximum swing flexion or gait symmetry could be suitable control target for the IPK.

## 1. Introduction

The loss of lower limb is usually caused by disease, trauma, and congenital disorder [1]. The way to restore walking ability is to install lower limb prosthesis [2]. A lower limb prosthesis generally consists of a socket, a knee joint, a pylon, and a prosthetic foot [3]. Because the knee needs to be stabilized and controlled by the amputee, the amputee's ability to walk safely and efficiently with the prosthesis is largely determined by the knee joint [4].

Prosthetic knee joints are currently described as mechanical or intelligent prosthetic (microprocessor-controlled) knees [5]. In general, mechanical control knees only provide swing or stance phase control with manual locking, constant friction, weight-activated friction, geometrically locking, pneumatics, or hydraulics [6]. They usually have no automated mechanism for adjustment when the walking speed or road condition changes. In contrast, intelligent prosthetic knees are equipped with sensors that continuously detect the position and the angular velocity of the prosthesis, as well as the forces that act on the ankle adapter [7]. This allows instantaneous adaptation of the flexion and extension resistance, which facilitates ambulation with varying walking speeds and cadence on different terrains, under various environmental conditions. The prosthetist can easily manipulate the control parameters of the intelligent prosthetic knee by means of software.

The energy cost, gait dynamics, and general mobility reflect the ability to perform gait tasks of the prosthetic knee [8]. Previous efforts have been made to develop prosthetic knee mechanisms that could increase stability in stance phase, flexibility, and gait symmetry during swing phase and, consequently, reduce the metabolic energy expenditure during gait. Indeed, one of the most important considerations in the design and prescription of lower limb prosthesis is the metabolic energy expenditure [9].

For amputee wearing an intelligent prosthetic knee, a physiological gait pattern may be realized with different control targets that could be generally divided into two groups, i.e., maximum swing flexion angle and gait symmetry. However, different control targets may lead to either reduced or increased energy costs in amputees. For control target as the maximum swing flexion angle, if the target angle is too large, the prosthetic knee joint will not be fully extended before the next heel strike. To prevent tripping under this condition, amputees are forced to either walk slower or work harder to push the knee forward during swing extension. Consequently, this may increase energy consumption and even cause uncomfortable gait patterns. On the other hand, gait symmetry may also be set as the control target, because a large difference in gait between the prosthesis and the amputee's contralateral limb may be visible and discordant. Asymmetry, or lack of symmetry, appears to be a relevant aspect for differentiating a normal and pathological gait. From control perspective, this can often be realized through the control of prosthetic knee to track the intact leg. And theoretically, gait symmetry may help reduce the metabolic costs in amputees.

A few previous studies exist that have compared the energy expenditure during ambulation of amputees wearing intelligent prosthetic knee and mechanically passive prosthetic knee. Datta et al. made the comparative evaluation of oxygen consumption in amputees using Intelligent Prosthesis and conventionally damped knee swing-phase control. Mean oxygen cost for all subjects at 0.69 m/s was 0.33 ml/kg.m with the conventional limb and 0.30 ml/kg.m with the Intelligent Prosthesis (p = 0.01). At 1.25 m/s the mean oxygen cost for the conventional limb was 0.24 ml/kg.m and for the Intelligent Prosthesis was 0.22 ml/kg.m. The results showed that oxygen cost of conventional limb and Intelligent Prosthesis decreased with the speed increased [10]. Jepson et al. assessed energy requirements using the Physiological Cost Index (PCI) to make a comparative evaluation of the Adaptive knee and Catech knee. The PCI results did not demonstrate improvement with the use of the Adaptive knee [11]. Johansson et al. compared the metabolic rate of two variable-damping knees, the hydraulic-based Otto Bock C-leg and the magnetorheological-based Ossur Rheo, with the mechanically passive, hydraulic-based Mauch SNS. When using the Rheo, metabolic rate decreased by 5% compared with the Mauch and by 3% compared with the C-leg. Metabolic cost during steady-state walking at a self-selected, comfortable speed was significantly different across the three tested knees. The results indicated that variable-damping knee prostheses offered metabolic energy expenditure advantages over mechanically passive designs for unilateral transfemoral amputees walking at self-selected ambulatory speeds [12]. Seymour et al. investigated energy expenditure between the C-leg and various nonmicroprocessor control (NMC) prosthetic knees. Statistically significant differences were found in oxygen consumption between prostheses at both typical and fast paces with the C-leg showing decreased values [13]. Kaufman et al. researched energy expenditure and activity of transfemoral amputees using mechanical and microprocessor-controlled prosthetic knees. Subjects demonstrated significantly increased physical activity–related energy expenditure levels in the participant's free-living environment after wearing the microprocessor-controlled prosthetic knee joint. There was no significant difference in the energy efficiency of walking [14]. However, all of these studies have primarily focused on the comparison between intelligent prosthetic knee joints and conventional mechanical prosthetic devices. The influence of control targets on metabolic energy expenditure of amputee using intelligent prosthetic knee is rarely known.

Therefore, the purpose of this study was to quantitatively compare the oxygen consumption in amputees wearing intelligent prosthetic knees when the control targets of intelligent knee are set to be maximum swing flexion and gait symmetry. The knowledge gained would help answer the following research question: whether different control targets in intelligent prosthetic knee may lead to different metabolic energy expenditures in amputees at different walking speeds?

## 2. Materials and Methods

### 2.1. Developed Intelligent Prosthetic Knee

**Figure 1:**
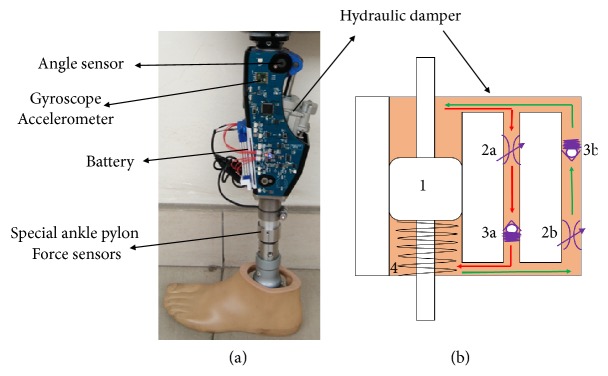
(a) Microprocessor-controlled knee prosthesis. (b) Functional principle of the hydraulic damper.

The intelligent prosthetic knee, shown in Figure 1(a), was designed based on the characteristics of hydraulic damping forces. The provided hydraulic system (Figure 1(b)) had two separate needle valves (2a, 2b) to generate joint resistance for the flexion and the extension movement. The valves opening were controlled by linear motors. As the valve opening changed, the flow resistance could be continuously varied from low to high values. When the piston 1 moved down during flexion, the oil flowed through flexion needle valve 2b and check valve 3b (flow marked in green). The steel spring was pressed during flexion by the displacement of the piston rod. For extension, the piston moved up and the oil passed extension needle valve 2a and check valve 3a (flow marked in red). The energy stored by compression of steel spring 4 was released. This could provide assistance for extension. Most of the sensors were integrated directly into the knee joint. In addition, loading sensors and ankle pressure sensor were built into the tube adapter that connected the knee joint with the prosthetic foot. Two prototypes of intelligent prosthetic knee had been made. They were mechanically identical except for the control targets. The control target of prototype one was maximum swing flexion and the other was the gait symmetry.

### 2.2. Control Target with Maximum Swing Flexion

**Figure 2:**
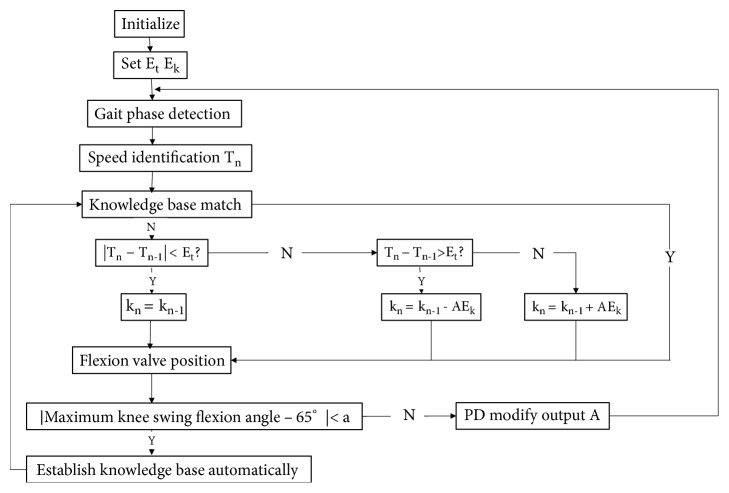
Control block diagram of swing flexion.

The auto-adaptation for swing flexion was designed to limit the maximum flexion angle for swing. The prosthetic knee joint and wearer were a nonlinear system [15]. Fuzzy logic control was easy to get good control in the nonlinear system with simple fuzzy inference [16]. Human walking was an unstable, strong coupling, and nonlinear system, which was suitable for fuzzy rules to control. The idea of control algorithm was to compare the differential of contact time for the stance phase in the sequential gait cycle with error threshold to control the valve position. The control block diagram of swing flexion was shown in Figure 2.

When the time error absolute value was less than the set value, it indicated that gait velocity had no change, and the valve position also kept the same with previous step:

  If |T_n_-T_n-1_| < E_t_ then K_n_ = K_n-1_;

When the time error was greater than the set value, it indicated that gait velocity decreased compared to forward step, and the valve position would decrease:

  If T_n_-T_n-1_ > E_t_ then K_n_ = K_n-1_ – A E_k_;

When the error is smaller than the negative set value, it indicated that gait velocity increased compared to forward step, and the valve position would increase:

  If T_n_-T_n-1_ < -E_t_ then K_n_ = K_n-1_ + A E_k_;

K_n_ is valve position calculated in the nth gait cycle; K_n-1_is valve position calculated in the (n-1)th gait cycle; T_n_-T_n-1_ is differential of contact time for the stance phase in the sequential gait cycle; A is gain coefficient; E_t_ is time error threshold; E_k_ is the minimum adjustment value of valve position; a: 5 degrees.

The gain coefficient A was adjusted through the fuzzy logic control. When the input error was larger, the bigger gain coefficient was used to increase the rate of convergence. When the input error was smaller, the lesser gain coefficient was used to ensure the stability of the control.

### 2.3. Control Target with Gait Symmetry

**Figure 3:**
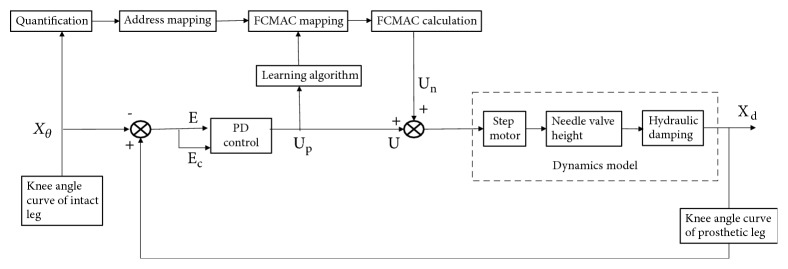
Control framework based on PD-FCMAC.

Cerebella model articulation controller (CMAC) neural networks were very suitable for real-time nonlinear system and had the advantage of fast learning characteristics [17]. The required storage capacity of CMAC control would has a geometric growth with the increase of input dimension. Thus, it affected the quantification of the input space series and limited the final study accuracy. To seek a better method of intelligent control of prosthetic knee, a hybrid inverse dynamic method based on PD and Fuzzy-CMAC (cerebellar model of fuzzy neural network) was proposed. The core concept of this method was making the prosthesis track the intact knee angle to realize gait symmetry [18]. The control framework was shown in Figure 3. It had two main characteristics: the feedforward control was realized through Fuzzy-CMAC and the feedback control was realized using traditional controller to ensure the stability of the system and inhibit the disturbance. The output signals *U*_*p*_ were obtained by cerebellar network feedback control through the PD controller and the input signals $X(\theta ,\dot{\theta },\ddot{\theta })$ were set for online training. PD/Fuzzy-CMAC had used the instructor *δ* learning algorithm. At the end of each control cycle, the corresponding Fuzzy-CMAC output *μ*_*n*_(k) was calculated. Then the total control input *μ*(*k*) was compared with *μ*_*n*_(k), and it could adjust the weight of the amendment into the learning process. The purpose of the learning was to make the difference smallest between the control input and the output of Fuzzy-CMAC. Adjust the target for the FCMAC by

(1) Ek=12μnk−μk2·1cΔωk=−η∂Ek∂ω=ημk−μnkcαi=ημpkcαiωk=ωk−1+Δωk+αωk−ωk−1

where *E*(*k*) was the error of controlling, *ω*(*k*) was weight value, *η* was network learning rate and *η* ∈ (0,1), *α* was inertial, and *α* ∈ (0,1).

At the beginning of the system run-time, let *ω* = 0, and then *μ*_*n*_ = 0, *μ* = *μ*_*n*_. At this point the system was controlled by the conventional controller. Through the learning of the Fuzzy-CMAC, the output of PD controller gradually became zero, and the output *μ*_*n*_(k) of CMAC control gradually converged to the total output *μ*(*k*).

### 2.4. Data Collection

Six transfemoral amputees gave informed consents to participate in this study. All subjects were surgically amputated due to trauma. The testing protocol was approved by the University of Shanghai for Science and Technology human subjects committee.

All subjects were recruited by the certified prosthetists in Shanghai. The inclusion criteria were (i) at least one year after amputation; (ii) functional level from K3 (i.e., the patient has the ability or potential for ambulation with variable cadence) or higher; (iii) never previously fitted with an intelligent prosthetic knee [19]. The six participants were 22-45 years old, 168-180 cm in height, and weighed 62-70 kg. The patient characteristics were summarized in Table 1.

All subjects were not permitted to drink alcohol or caffeine for 24 hours prior to testing. The subjects' diets were recorded on the day of and prior to the testing session. The similar diets were carried out for the following test.

The Group 1 experiments were performed with the subjects wearing the knee prosthesis that had control target of maximum swing flexion (described as MSF). Each individual was given approximately 5 hrs to adapt to the wearing of the knee prosthesis. Before the test began, the subjects were requested to practice walking on a treadmill that had a 1.8 × 1.2 m^2^ surface area. When a normal gait pattern was observed by the prosthetist, the subject was allowed to have a rest for about 20~30 mins. The subject was then requested to walk consecutively on the treadmill at the specific walking speeds for a total of 19 minutes. The first 2 minutes were for the warm-up, followed by five sessions at different walking speeds (3min walking at 0.5m/s, 3 min at 0.7m/s, 3 min at 0.9m/s, 3 min at 1.1m/s, and 3 min at1.3m/s). The last 2 minutes were for the subject to slow down. To obtain oxygen consumption data, subjects wore a mouthpiece and nose plug to collect gases during tests. Through this period, breath-by-breath analysis of the subject's expired air was carried out by means of Ultima™ CardiO_2_® (MGC Diagnostics Corporation, USA) gas exchange analysis system. Oxygen consumption was normalized to body weight (milliliter O_2_/kilogram/minute) for each testing trial.

The Group 2 experiments were conducted four weeks later. The same experimental procedure was repeated except that the prosthetic knee had control target of gait symmetry (described as GS). When the control target was the GS, the prosthetic knee tracked the joint angle from the contralateral knee during walking. To achieve this target, a knee angle sensor was placed on contralateral leg of the subject to serve as an input signal to the prosthetic knee. In all cases, the same socket was used in both trials and only the prosthetic knees were changed for the MSF and GS trials. The fitting and alignment of the prosthetic knee to all six subjects were carried out by the same prosthetist.

## 3. Results and Discussion

### 3.1. Results

**Figure 4:**
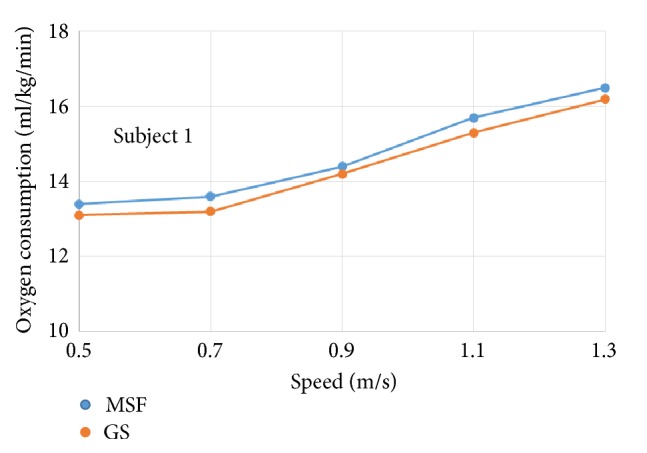
Oxygen consumption under different speeds for subject 1.

The oxygen consumption for individual subject wearing prosthetic knees of different control targets was plotted against increasing walking speeds, respectively (see Figures 4~9).

The six subjects did not show statistically significant differences in oxygen consumption when the control target was MSF compared with the GS. There were general trends that the oxygen consumption increased with the increased walking speeds, regardless of the control targets. The ANOVA tests showed that the overall effects of the control targets on oxygen consumption were not significant across all walking speeds (Table 2). However, individual testing results showed that oxygen consumption for subjects 1, 4, and 6 were generally lower when the control target was GS under given testing speed. In contrast, oxygen consumption for subjects 3 and 5 was lower when the control target was MSF under given testing speed. Subject 2 showed mixed effects on walking efficiency across different speeds.

### 3.2. Discussion

Our results clearly demonstrated that the net oxygen consumption increased as the walking speed increased when the amputees used the intelligent prosthetic knee, no matter the control target was MSF or GS. It was different with the previous report by Datta et al. [10]. Although the focus of their study was comparative evaluation of oxygen consumption in amputees using Intelligent Prostheses and conventionally damped knee, their results showed that the mean oxygen consumption decreased with the increased walking speed.

Previous study suggested that the oxygen consumption (ml/kg/min) for able-bodied individuals during level-ground walking could be predicted using the formula VO_2_ = 0.1 *∗* speed (m/min) + 3.5 [13]. Using the above formula, oxygen consumption prediction for the subjects should increase with the increased walking speed. The trend of our results was in line with the formula. However, the oxygen consumption for our subjects was generally higher than those predicted by the formula. The reason might be that the formula was based on data obtained from healthy people walking on the level ground, while the current tests were for amputees wearing prosthetic knee walking on the treadmill.

Our study also demonstrated that the control targets of maximum swing flexion or gait symmetry showed no significant difference in oxygen consumption over a range of walking speeds. This might explain why many researches had chosen the maximum swing flexion or gait symmetry to be the performance contrast indicators of prosthetic knees. Prinsena et al. compared the Rheo Knee II (a microprocessor-controlled prosthetic knee) with NMPKs across varying walking speeds. No differences on maximum swing flexion were found between prosthetic knee conditions. In addition, maximum swing flexion knee angle increased significantly with walking speed for both prosthetic knee conditions [19]. Julius et al. showed that the slope of the linear regression line of the maximum swing flexion under increased walking speed was 3.5°/m/s with C-Leg, 28.1°/m/s with Plié2.0, 18.3°/m/s with Orion, and 17.0°/m/s with Rel-K. On the contralateral side, the natural knee flexion angle was similar with all tested knee joints, resulting in a mean slope of 6.2°/m/s [20]. Kaufman et al. compared the gait symmetry of active transfemoral amputees while using a passive mechanical knee joint or a microprocessor-controlled knee joint. The results showed that the use of the microprocessor-controlled knee joint resulted in improved gait symmetry. These improvements might lead to a reduction in the degenerative musculoskeletal changes often experienced by amputees [21]. The choice of performance contrast indicators of maximum swing flexion or gait symmetry seemed to be supported by the results of this work.

The oxygen consumption was similar to previous research with other prosthetic knees. In the research of Seymour et al., mean oxygen consumption with C-leg was 12.6 ± 1(ml/kg/min) in typical pace (49 ± 15m/min) and 16.0 ± 2(ml/kg/min) in fast pace (70 ± 20m/min) [13]. Although the results in this work were a little higher, the differences were acceptable.

This study had several limitations. A number of confounding factors might have contributed to the limited differences we found. The sample size was small. It affected statistical power and thereby the ability to detect significant differences. The tests were all level-walking. More realistic conditions including uneven terrain, sitting down, and standing up rather than steady level walking may be more revealing.

## 4. Conclusions

The aim of the present work was to find out the metabolic energy expenditure difference of amputees using IPK with control targets of MSF and GS and determine which target was more suitable for the control of IPK based on the metabolic energy expenditure assessment. We concluded that the control targets of maximum swing flexion and gait symmetry had no significant difference on metabolic energy expenditure of amputee using intelligent prosthetic knee. From perspective of amputee's metabolic costs, either maximum swing flexion or gait symmetry could be suitable control targets for IPK. No matter the control target of IPK was maximum swing flexion or gait symmetry, the oxygen consumption increased with the increased walking speed. The trend of the results was in line with able-bodied individuals walking over level ground.

## Figures and Tables

**Figure 5:**
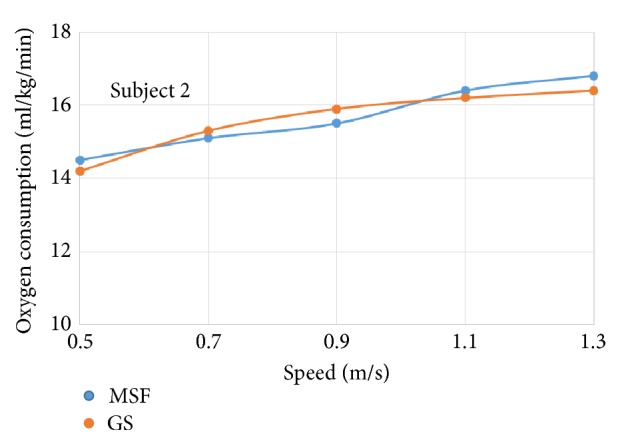
Oxygen consumption under different speeds for subject 2.

**Figure 6:**
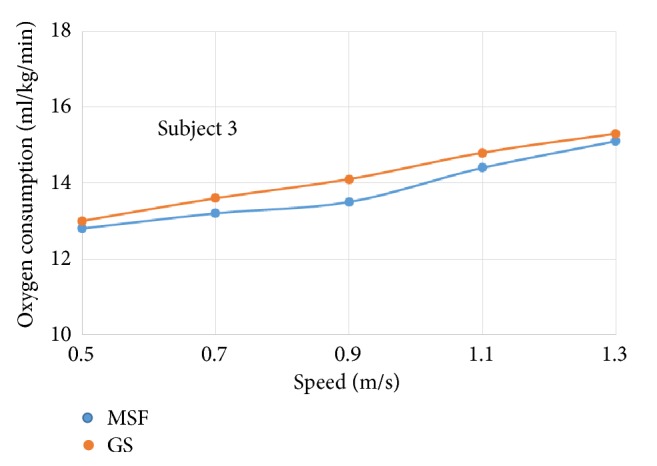
Oxygen consumption under different speeds for subject 3.

**Figure 7:**
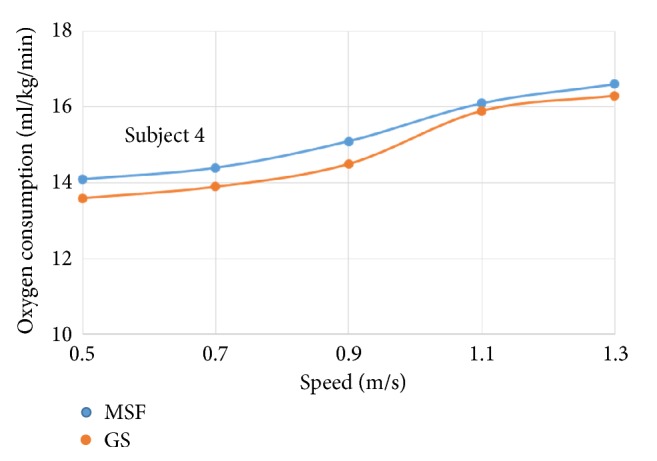
Oxygen consumption under different speeds for subject 4.

**Figure 8:**
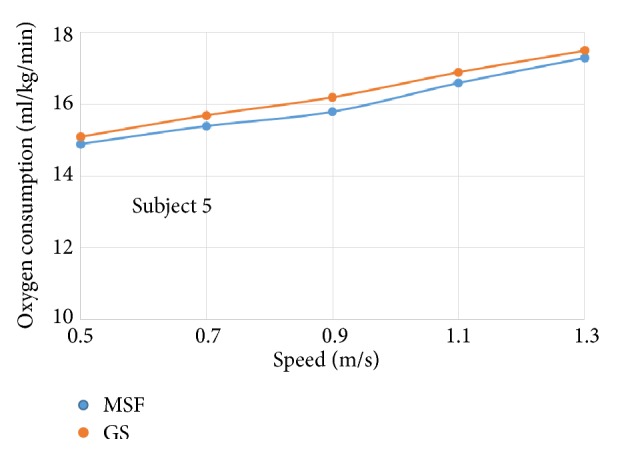
Oxygen consumption under different speeds for subject 5.

**Figure 9:**
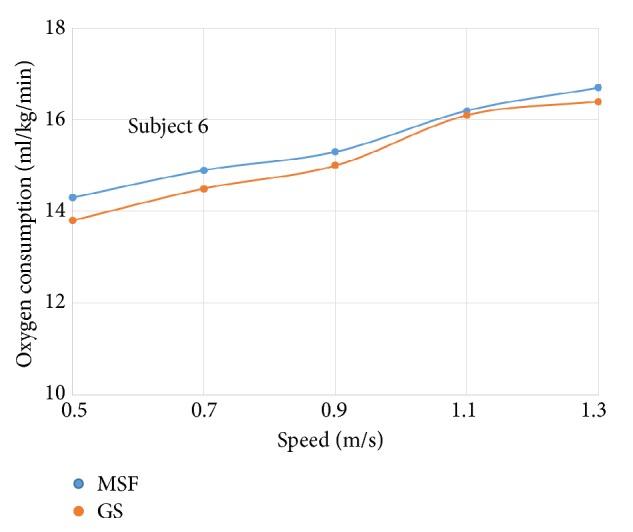
Oxygen consumption under different speeds for subject 6.

**Table 1:** Subject demographics.

Subject	Age(years)	Height(cm)	Weight(kg)	Gender	K-Level
1	22	175	70	Male	K4
2	42	173	70	Male	K3
3	35	180	75	Male	K4
4	37	168	62	Male	K3
5	45	176	72	Male	K3
6	40	173	63	Male	K3

**Table 2:** Mean comparisons of oxygen consumption.

Speed (m/s)	Oxygen consumption(ml/kg/min)		P-value
	MSF	GS	
0.5	14 ± 0.77	13.8 ± 0.78	0.664
0.7	14.43 ± 0.87	14.37 ± 0.98	0.904
0.9	14.93 ± 0.85	14.98 ± 0.89	0.922
1.1	15.9 ± 0.79	15.87 ± 0.73	0.941
1.3	16.5 ± 0.74	16.35 ± 0.7	0.726

## Data Availability

The data used to support the findings of this study are available from the corresponding author upon request.
